# Metabolomic profiling in blood from umbilical cords of low birth weight newborns

**DOI:** 10.1186/1479-5876-10-142

**Published:** 2012-07-09

**Authors:** Carmen Ivorra, Consuelo García-Vicent, Felipe Javier Chaves, Daniel Monleón, José Manuel Morales, Empar Lurbe

**Affiliations:** 1 Cardiovascular Risk Unit, Consorcio, Hospital General, University of Valencia, Av. Tres Cruces s/n, Valencia 46014, Spain; 2 CIBER Fisiopatología Obesidad y Nutrición (CB06/03), Instituto de Salud Carlos III, Madrid, Spain; 3 Unidad de Genotipado y Diagnóstico Genético, Fundación Investigación, Hospital Clínico Universitario de Valencia/INCLIVA Valencia, Av. Blasco Ibáñez, 17, Valencia, 46010, Spain; 4 CIBER de Diabetes y Enfermedades Metabólicas (CIBERDEM), Madrid, Spain; 5 Fundación Investigación Hospital Clínico Universitario de Valencia / INCLIVA, Av. Blasco Ibáñez, 17, Valencia, Spain; 6 Unidad Central Investigación Medicina, Universidad de Valencia /INCLIVA, Av. Blasco Ibáñez, 17, Valencia, 46014, Spain; 7 Department of Pediatrics, Consorcio Hospital General, University of Valencia, Av. Tres Cruces s/n, Valencia, 46014, Spain

**Keywords:** Low birth weight, Umbilical cord, Metabolomics, Amino acids, Fetal factors

## Abstract

**Background:**

Low birth weight has been linked to an increased risk to develop obesity, type 2 diabetes, and hypertension in adult life, although the mechanisms underlying the association are not well understood. The objective was to determine whether the metabolomic profile of plasma from umbilical cord differs between low and normal birth weight newborns.

**Methods:**

Fifty healthy pregnant women and their infants were selected. The eligibility criteria were being born at term and having a normal pregnancy. Pairs were grouped according to their birth weight: low birth weight (LBW, birth weight < 10^th^ percentile, n = 20) and control (control, birth weight between the 75^th^-90^th^ percentiles, n = 30). Nuclear Magnetic Resonance (NMR) was used to generate metabolic fingerprints of umbilical cord plasma samples. Simultaneously, the metabolomic profiles of the mothers were analysed. The resulting data were subjected to chemometric, principal component and partial least squares discriminant analyses.

**Results:**

Umbilical cord plasma from LBW and control newborns displayed a clearly differentiated metabolic profile. Seven metabolites were identified that discriminate the LBW from the control group. LBW newborns had lower levels of choline, proline, glutamine, alanine and glucose than did the control newborns, while plasma levels of phenylalanine and citrulline were higher in LBW newborns (p < 0.05). No significant differences were found between the two groups of mothers.

**Conclusions:**

Low birth weight newborns display a differential metabolomic profile than those of normal birth weight, a finding not present in the mothers. The meaning and the potential utility of the findings as biomarkers of risk need to be addressed in future studies.

## Background

The concept of the fetal origin of adult disease suggests that early life conditions can “program” the fetus for a spectrum of adverse health outcomes in adulthood [1,2]. Low birth weight babies (LBW), typically defined as birth weight below the 10^th^ percentile according to gestational age [3], have an increased risk of rapid postnatal weight gain, later obesity, and diseases in adulthood such as type 2 diabetes, hypertension and ischemic heart disease [4-6]. Therefore, cardiovascular risk is determined not only by conventional risk factors of importance in adult life, but also by early life programming based on intrauterine fetal growth [7].

The mechanistic pathways underlying the relationship between fetal growth restriction and cardiovascular risk are poorly understood [8]. Although the cause of this relationship is unknown, several hypotheses have been proposed: involvement of the kidney (reduction of nephron number, activation of the renin-angiotensin system, an increase in renal sympathetic nerve activity), the neuroendocrine system (up-regulation of the hypothalamic-pituitary-adrenal axis, altered adaptation to stress), and early abnormalities in the vasculature tree [9,10].

A previous study has demonstrated that endothelial cells from umbilical cord artery derived from individuals of LBW exhibit a different phenotype when compared to that from individuals of higher birth weight [11]. This observation could imply that it is possible to identify functional and/or structural differences at birth which can contribute to providing better information with respect to the risk of developing disease later in life. A further approach to studying differences between low and normal birth weight newborns is to assess their metabolomic profiles.

Metabolomics, the study of small-molecule metabolites, is a discipline that focuses on the measurement of the relative concentrations of endogenous small molecules in biofluids. This technology may be useful for understanding metabolic imbalances and for diagnosing human diseases. Recently, studies using metabolomics have been used to identify a discriminatory metabolite profile in a number of areas: preeclampsia in early pregnancy [12], diabetes [13], obesity [14], [15], risk for preterm delivery [16]. It has been suggested they predict the presence and severity of cardiovascular disease [17-20]. There are no data about its use to identify a metabolite profile in umbilical cord from LBW children. The objective of the present study was to analyse the metabolomic profile at birth in newborns with LBW, and to compare it with those from normal birth weight children. Simultaneously, the metabolomic profiles of their mothers were analysed. The identification of deregulated metabolites would potentially give clues for better understanding the abnormalities associated with LBW.

## Methods

### Subjects and sample collection

Newborns were included if born at term (gestational age ≥37 weeks), after uncomplicated pregnancies ascertained according to the Ballard method [21], and by normal delivery or Cesarean section in the absence of perinatal illness at the Hospital General Universitario of Valencia, Spain. All the mothers were healthy and had no cardiovascular risk factors. Fifty newborns and their mothers of European origin were selected to participate in the study. Umbilical cord blood samples were obtained from the clamped umbilical cord immediately after delivery. The samples of their mothers were drawn at 2–4 h after delivery in non-fasting conditions, but with at least 8 hours having passed since the last meal and blood sampling. Two groups of newborns were created according to birth weight: lower than the 10^th^ percentile (lower than 2.5 kg; LBW group, n = 20) or appropriate birth weight for gestational age (control group, birth weight between the 75^th^-90^th^ percentiles, n = 30) [22]. Parents gave their consent for the study after they were given informed in writing of the objectives of the research project and of the samples that would be taken. The study was approved by the hospital’s review board and was carried out in accordance with the Declaration of Helsinki. Both the samples (plasma from umbilical cord of the newborns and plasma from peripheral veins from the mothers), as well as the collected data, were stored according to the directives dictated by the law of Biomedical Investigation of 2007 (Law 14/2007) and all applicable rules.

### Metabolomic profile

#### Storage, preparation and ^1^ H Nuclear magnetic resonance spectroscopic analysis of blood plasma

Umbilical venous cord blood and maternal peripheral venous blood were collected in EDTA-tubes, centrifuged to yield plasma, stored at −80 °C and thawed before use. For Nuclear Magnetic Resonance (NMR) analysis, 500 μl of plasma were mixed with 50 μl of D_2_O (as a field lock). A total of 500 μl of the mixture of each sample was then individually transferred into a 5 mm high quality NMR tube. All ^1^ H NMR spectra were acquired using a standard one-dimensional pulse sequence with water suppression (Bruker Avance 600 spectrometer operating at 600.13 MHz with a 1 mm ^1^ H/^13^ C/^15^ N TXI probe). A total of 256 FIDs (free induction decay) were collected into 64 k data points with a spectral width of 14 ppm and a recycle delay (RD) of 1 s. The water signal was saturated with weak irradiation during the recycle delay. Before Fourier transformation, the free induction decay was multiplied by a 0.3 Hz exponential line broadening.

Spectral chemical shift referencing on the Alanine CH_3_ doublet signal at 1.475 ppm was performed in all spectra. Resonances in these spectral regions were assigned using the literature [23] and two-dimensional spectra were selected (2D NMR, especially for long chain metabolites). Spectral regions between 0.5 and 4.5 ppm and between 5.5 and 9.5 ppm were binned in segments of 0.01 ppm width (6 Hz) for multivariate analysis. Data binning in NMR spectra for multivariate analysis is a common pre-processing practice [24,25], which, although it may decrease the effect of minor observations, allows for the detection of relevant regions, decreases the risk of model overfitting and artificially increases the signal to noise-ratio. Data binning reduces the impact of read noise on the processed data at the cost of a lower resolution. We used binning only for the pattern recognition. There are two benefits associated with the use of pattern recognition approaches in the binning of spectral data. First, the signal-to-noise ratio increases. Second, there is a reduction in the possibility of overfitting. Once the bucket with the relevant peaks in each spectra were identified, they were quantified. Therefore, the values reported are quantified peaks by spectral integration. The binned data was normalised to the total spectral area. Thus, the individual peak intensities were normalized to total metabolite content for better a comparison between samples. Spectral regions belonging to the EDTA resonances (2.52 to 2.57 ppm and 3.06 to3.17 ppm) were removed from the spectra for subsequent analysis. The available spectral databases and 2D NMR experiments were used to aid in structural identification of the relevant metabolites. All spectra were processed using Topspin 1.3 (Bruker Biospin GmbH, Germany) and transferred to MATLAB® (MathWorks Inc, 2006) using in-house scripts for data analysis. Signals belonging to selected metabolites were integrated and quantified using semi-automated in-house MATLAB peak-fitting routines. These fitting routines were based on Levenburg-Marquard optimization procedures. The target function for the optimization included experimental spectra measured for standard solutions of selected metabolites with complex multiplet patterns and theoretically generated Lorentzian-shape signals for those metabolites with simpler spectral patterns. One way analysis of variance (ANOVA) was used for the determination of statistical significance between group means of the corresponding integrals.

### Multivariate analysis of nuclear magnetic resonance spectra

In the present study, binned spectral regions for blood plasma were mean-centered chemometric analyses. Relevant bins were identified in our chemometric models. The relevant spectral regions within these bins were identified and quantified by spectral integration. Chemometric and statistical analyses were performed using in-house MATLAB scripts and the PLS Toolbox (Eigenvector Research, Inc.). Principal Component Analysis (PCA) and Projection to Latent Structures for Discriminant Analysis (PLS-DA) were applied to NMR spectra data sets. PCA is able to find low dimensional embeddings of multivariate data in a way that optimally preserves the structure of the data. The PCA technique transforms the variables of a data set into a smaller number of new latent variables called principal components (PCs) which are uncorrelated to each other and account for decreasing proportions of the total variance of the original variables. Each new PC is a linear combination of the original variation so that a compact description of the variation within the data set is generated. Observations are assigned scores according to the variation measured by the principal component X, with those having similar scores clustering together. Where PCA proved inadequate to define clustering, a supervised approach was used.

PLS-DA is a classification technique that combines the properties of Partial Least Squares regression with the discrimination power of discriminant analysis [26]. The main advantage of PLS-DA models is that the main sources of variability in the data are modeled by the so-called latent variables (LVs) and, consequently, in their associated scores and loadings. This allows for the visualization and understanding of different patterns and relations in the data. PLS-DA is a supervised extension of PCA that is used to distinguish two or more classes by searching for variables (X matrix) that are correlated to class membership (Y matrix). In this approach, the axes are calculated to maximize class separation and can be used to examine separation that would otherwise be across three or more principal components [26]. In order to discriminate between samples associated with low weight and normal weight at birth, a PLS-DA model was devised. The PLS-DA model discriminating between controls and LBW was cross-validated by technical replication of the whole process on 25 random data splits. To validate the model, important quality parameters were calculated: R^2^X, R^2^Y, Q^2^ and RMSP. R^2^X and R^2^Y describe the variation in the X and Y variables, respectively. Q^2^ describes predictability. RMSP (Root mean squared error of prediction) gives a direct indication of the error in the predicted value.

### Statistical analysis

Values were expressed as mean ± SD for each study group. The differences in the mean values of anthropometric parameters between the groups were assessed by analysis of variance (ANOVA) adjusted by sex. The normality of the data was verified using the Kolmogorov-Smirnov test. Choline, glutamine, alanine and glycogen in the control group do not have a normal distribution, and a Kruskal-Wallis test was used for these variables**.** An outlier analysis was performed, and those samples deviating from the mean by more than 2.5 times the standard deviation were excluded. A significant difference was considered present if p < 0.05. Pearson’s correlation coefficients were used to examine the relations between variables. Statistical analyses were performed using SPSS 15.0. (SPSS Inc, Chicago, Illinois, USA) and GraphPad Statmate 5.0 (GraphPad Software, La Jolla, California, USA) software graphs.

## Results

### Clinical characteristics of mothers and newborns

The general characteristics of mothers and their newborns are shown in Table 1. No differences were observed in maternal age, maternal BMI, pregnancy weight gain or type of delivery between the two groups. Although no significant differences were observed in heart rate and diastolic blood pressure between newborns groups (Table 1), differences appeared in sex, height, head circumference and blood pressure. As expected, the LBW group had systolic values significantly lower than those for the control birth weight group [10]. All comparisons between newborns groups were adjusted by sex, and no changes were noted when comparing boys with girls.

**Table 1:** Anthropometric and clinical characteristics of mothers and newborns grouped by birth weight

	**Control (n = 30)**	**LBW (n = 20)**	**p-value**
**Maternal Characteristics**			
Maternal age (years)	30.22 ± 5.65	30.50 ± 6.10	NS
Pregnancy weight gain (g)	12581 ± 428	11025 ± 301	NS
Delivery (vaginal/Cesarean section)	21/9 (70%)	12/8 (60%)	NS
Maternal Body Mass Index	24.0 ± 3.1	22.8 ± 2.1	NS
**Newborns Characteristics**			
Gestational age at delivery (weeks)	38.95 ± 0.90	37.64 ± 1.50	p < 0.05
Sex (male/female)	20/10 (66.7%)	5/15 (33.3%)	p < 0.01
Birth weight (g)	3756 ± 148	2371 ± 177	p < 0.0001
Height (cm)	50.46 ± 1.45	45.55 ± 1.14	p < 0.0001
Head circumference (cm)	34.70 ± 1.00	32.10 ± 1.80	p < 0.0001
Systolic blood pressure (mmHg)	73.88 ± 13.20	65.15 ± 11.50	p < 0.05
Diastolic blood pressure (mmHg)	44.20 ± 11.22	40.70 ± 8.70	NS
Heart rate (bpm)	130.14 ± 16.30	128.33 ± 17.90	NS

The values are expressed as mean ± SD; p-value, statistical significance of the differences among groups; NS, Non-significant; LBW, low birth weight.

### Metabolomic profile in umbilical cord blood in low birth weight and control newborns

**Figure 1:**
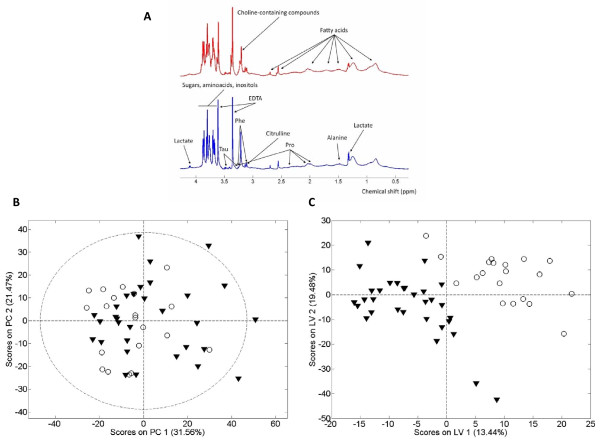
** Umbilical cord blood profile. **Superposed ^1 ^H NMR spectra for the spectra of umbilical cord blood plasma (Panel **A**), Principal Component Analysis scores plot (PCA, panel **B**), and Projection to Latent Structures for Discriminant Analysis scores plot (PLS-DA, panel **C**) showing the global metabolic differences between low birth weight (open circles, blue spectra) and control birth weight (black triangles and red spectra) newborns. Although an unsupervised analysis by PCA does not show differences between groups, a supervised classification method (PLS-DA) provides a differential global metabolic profile. The cross-validated error percentage of the model for the classification of low weight at birth samples is 8%. Metabolites exhibiting largest differences between groups have been listed in Table 2.

The standard 1D NMR spectrum is shown in Figure 1A. PCA and PLS-DA scatter plot were performed on the pre-processed NMR spectra comparing plasma from LBW versus control newborns and were used to identify metabolite clusters. Although an unsupervised analysis by PCA does not show differences between groups, Figure 1B, a supervised classification method (PLS-DA, 3 Latent Variables, see Additional file 1: Figure S1) provides a differential global metabolic profile, Figure 1C. The model had a R^2^X (cumulative) of 0.623, a R^2^Y (cumulative) of 0.871, a Q^2^ (cumulative) of 0.523 (a Q^2^ value superior to 0.5 is generally considered to be a good predictor) and a RMSP of 0.417 (medium predictive capacity). Spectral regions and peaks with the highest contribution to the loadings plot from this model were further quantified and analysed. Many metabolites may contribute to these loading plots and to the separation between classes. A total of thirty-four metabolites were tested in fifty-eight spectral regions, and the differences were evaluated by the T-Student test with the Kruskal-Wallis correction. However, only those that differed with p < 0.05 were taken into consideration.

Of the 34 metabolic regions studied, 17 compounds were quantified according to the loadings of the multivariate analysis. Among the 17 metabolites, significant differences were observed for seven (proline, free choline, glutamine, alanine, glucose, phenylalanine and citrulline) between the LBW and the control group, while no differences were observed in the other seven (spectral region containing lipoproteins peaks, in carbohydrates and glycogen fragments, in ketone bodies like acetoacetate and hydroxybutyrate, in total lipids and in fatty acids both saturated and unsaturated) (data not shown).

**Figure 2:**
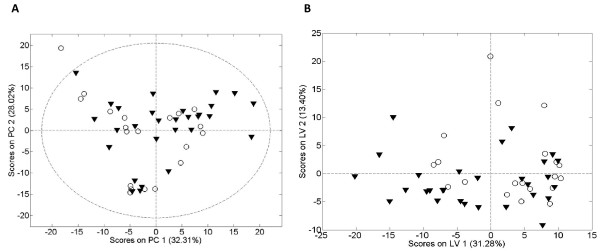
** Metabolites distinguishing LBW and control newborns. **Glucose, Proline, Glutamine, Choline, and Alanine levels were higher in control newborns. Significantly higher levels of citrulline and phenylalanine were detected in umbilical cord plasma from LBW newborns. Mean metabolite abundance is indicated by line. Each point represents a single newborn.

Table 2 shows median values ± SD for arbitrary units of intensity (AUIx10^-3^) from identified metabolites, and Figure 2 provides the individual levels of 7 metabolites that display significant level differences between the two groups. In the LBW group, all metabolites have a normal distribution, and samples are more homogeneous than in the control group. In the control group, choline, glutamine and alanine do not have a normal distribution. An outlier analysis was carried out, and two subjects were excluded from the control group.

**Table 2:** List of identified metabolites in umbilical cord plasma and in maternal peripheral blood

	**NEWBORNS**		**p-value**	**MOTHERS**		**p-value**	**Feto-maternal ratio**		**p-value**
	**Control (n = 30)**	**LBW (n = 20)**	**C vs LBW**	**M-Control (n = 30)**	**M-LBW (n = 20)**	**M-C vs M-LBW**	**Control**	**LBW**	**Control vs LBW**
**Amino Acids and related**									
Proline	21.74 ± 9.80	13.00 ± 7.50	0.002	12.22 ± 0.92	12.10 ± 0.56	NS	1.73 ± 0.83	1.26 ± 0.62	0.023
Free Choline	3.76 ± 0.17	2.58 ± 0.07	0.005	5.73 ± 0.79	5.80 ± 0.76	NS	0.56 ± 0.17	0.46 ± 0.17	0.040
Citrulline	0.67 ± 0.02	0.84 ± 0.02	0.008	15.16 ± 1.09	15.34 ± 1.22	NS	0.04 ± 0.01	0.05 ± 0.02	0.022
Glutamine	3.68 ± 0.17	2.59 ± 0.07	0.004	0.85 ± 0.06	0.85 ± 0.07	NS	3.68 ± 1.16	3.07 ± 0.81	0.024
Alanine	9.49 ± 4.10	7.00 ± 2.20	0.008	0.90 ± 0.03	0.96 ± 0.20	NS	9.28 ± 3.29	7.66 ± 2.64	0.041
Phenylalanine	1.07 ± 0.03	1.27 ± 0.03	0.018	9.78 ± 1.13	9.97 ± 0.96	NS	0.12 ± 0.03	0.13 ± 0.03	NS
**Carbohydrates**									
Glucose	2.15 ± 0.98	1.59 ± 0.50	0.023	1.12 ± 0.08	1.10 ± 0.09	NS	1.65 ± 0.58	1.43 ± 0.42	NS
Glycogen fragments	2.70 ± 1.11	2.01 ± 0.76	0.011	2.95 ± 0.33	2.82 ± 0.24	NS	0.81 ± 0.24	0.71 ± 0.27	NS
**Lipids**									
LDL + VLDL enriched spectral region	7.22 ± 1.25	7.60 ± 0.99	NS	5.01 ± 0. 50	4.96 ± 0.52	NS	1.52 ± 0.33	1.55 ± 0.23	NS
PUFA	N/D	N/D	--	4.19 ± 0.49	4.38 ± 0.38	NS	--	--	--

The values are expressed as mean ± SD; p-value, statistical significance of the differences among groups; N/D, not detected; LBW, low birth weight. Mean metabolite abundance is indicated by arbitrary units of intensity (AUIx10^-3^).

Five metabolites (proline, choline, glutamine, alanine and glucose) were reduced in the LBW group as compared to those for the controls. In the LBW group, proline values were 38.1% less (21.74 ± 9.80 AUI vs 13.00 ± 7.5 AUI; p-value = 0.002) while free choline was 31.4% less (3.76 ± 0.17 AU vs 2.58 ± 0.07 AU; p-value = 0.005). Glutamine (3.68 ± 0.17 vs 2.59 ± 0.07; p-value = 0.004), alanine (9.49 ± 4.1 vs 7.00 ± 2.2; p-value = 0.008) and glucose levels (2.15 ± 0.98 vs 1.59 ± 0.50; p-value = 0.023) were significantly lower in LBW newborns as well. In addition, LBW newborns exhibited a 15.7% increase in values of phenylalanine (1.07 ± 0.03 vs 1.27 ± 0.03 p-value = 0.018) and a 20.2% increase in values of citrulline (0.67 ± 0.02 vs 0.84 ± 0.002; p-value = 0.008). No difference in profiles between sexes was observed.

### Maternal metabolic profile in venous peripheral blood

**Figure 3:**
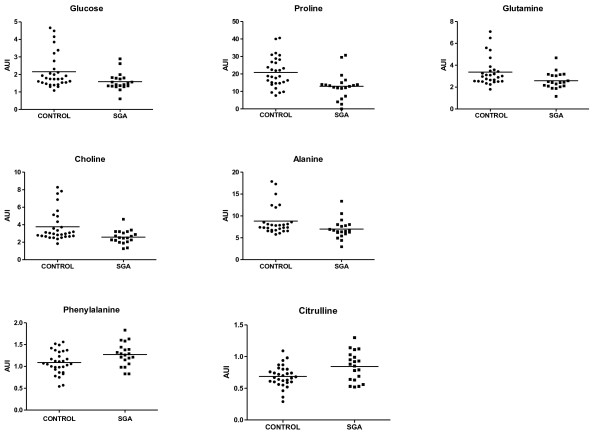
** Spectra of maternal venous plasma. **Principal Component Analysis (PCA, panel **A**) and Projection to Latent Structures for Discriminant Analysis (PLS-DA, panel **B**) scores plot for the spectra of maternal venous plasma showing the global metabolic differences between low birth weight (open circles) and control birth weight (black triangles ) newborns. Apparently, there are no global metabolic trends in the maternal blood associated with the weight of the newborns.

Nuclear Magnetic Resonance data from mothers were processed to give the initial PCA plot after a partial least squares-discriminate analysis (Figure 3, PLS-DA, 3 Latent Variables, see Additional file 1: Figure S2). The PLS-DA model had a R^2^X (cumulative) of 0.512, a R^2^Y (cumulative) of 0.611, a Q^2^ (cumulative) of 0.502 (a Q^2^ value superior to 0.5 is generally considered to be a good predictor) and a RMSP of 0.282 (low predictive capacity). The loadings plot showed 14 spectral regions with high relative weight belonging to 10 metabolites. Table 2 shows median values ± SD for arbitrary units of intensity (AUIx10^-3^) from the identified metabolites. From a total of 10 metabolites, 6 were related to the amino acid group (proline, phenylalanine, alanine, glutamine, citrulline and free choline), 2 were related to the carbohydrate group (glucose and glycogen fragments), and 2 to the lipid group (spectral region containing large LDL + VLDL lipoproteins peaks and polyunsaturated fatty acids (PUFAs)). The results of the metabolomic profiling in maternal blood showed no significant differences between the two groups of mothers at either the PLS-DA score plot level and at the individual identified metabolite level (Table 2, p-value M-C vs M-LBW).

The feto-maternal ratio for carbohydrates (glucose and glycogen fragments), lipids (spectral region containing large LDL + VLDL lipoprotein peaks) and phenylalanine was comparable between the two groups (Table 2, p-value Control vs LBW). In contrast, the feto-maternal ratio for proline, free choline, glutamine and alanine was significantly lower (p < 0.05) in the LBW group, whereas that for citrulline was significantly higher.

### Relationship between metabolites and birth weight

Pearson correlations were performed for each of the 7 aforementioned metabolites versus birth weight. Out of the 7 metabolites, the analysis identified a significant positive correlation between birth weight and free choline, proline, and glutamine (free choline r = 0.50, p-value <0.01; proline r = 0.29, p-value < 0.05; glutamine r = 0.36, p-value < 0.01). A significant inverse association was found between citrulline and birth weight (r = −0.40, p-value <0.01) and between phenylalanine and birth weight (r = −0.34, p-value <0.05); i.e., phenylalanine and citrulline increase as birth weight decreases. No relationships were identified when correlating these metabolites with blood pressure. No significant associations were found between alanine and glucose with birth weight.

## Discussion

The present study is the first to utilize metabolomic analysis in umbilical cord blood samples from LBW children in order to identify metabolites that could be related to the abnormalities associated with the long-term development of the increased cardio-metabolic risk associated with LBW. The metabolomic profile of LBW newborns differed from that observed in a group of newborn controls. Higher values of phenylalanine and citrulline and lower levels of proline, choline, glutamine, alanine and glucose were observed. A significant relationship between some of these metabolites and birth weight was also present. Furthermore, the differences observed in the newborns were not observed in their mothers, all of whom were healthy and had similar weight gains during pregnancy. Although the significance of the observed metabolomic values is not well understood, interesting information can be derived from the present results since some of the observed data have been linked previously to the abnormal metabolic states associated with LBW.

Even though the impact of fetal metabolic programming on adult health is well documented, the underlying mechanisms are poorly understood. Animal models have been used extensively to investigate the mechanism by which the early-life environment induces persistent alterations in the metabolism of offspring [27-30]. These studies support the hypothesis that nutritional imbalance during prenatal and early postnatal life can induce long term metabolic changes and increase susceptibility to metabolic diseases in later life [31-33].

The absence of differences found for lipoproteins, carbohydrates, ketone bodies and fatty acids suggests that fatty acid oxidation and glucose metabolism remain unaltered. Changes, however, in the spectral region between 7.27 and 5.36 ppm, which has been assigned to glycogen fragments, may suggest alterations in carbohydrate storage. The large molecular size of glycogen makes it very difficult to observe its ^1^ H resonance signals. Longer NMR T_2_ relaxation times, which suggest smaller molecule sizes, produce the narrow signals observed for that spectral region. Perhaps some macromolecule degradation of glycogen into smaller molecular size fragments is the cause.

In the present study, the mismatch between the mother and the newborn in terms of the metabolomic profile is worth taking into consideration. While a different pattern was observed in the LBW newborns compared to controls, the mothers of both groups had a similar pattern. A trend towards reduced levels of amino acids in the umbilical cord was observed in LBW, something not observed in the mothers. The reason for the discrepancies is not well known, although suboptimal placental transport of amino acids could be involved. Impaired placental amino acid transport is often seen in association with IUGR, yet it is unclear whether the changes in amino acid transport are a cause or a consequence of IUGR [34,35]. Experimental models in rats, however, have suggested a down-regulation of amino acid transporters as a cause rather than a consequence [36]. Consistent with impaired placental amino acid transport as a potential cause of IUGR in humans, umbilical cord blood (but not maternal blood) concentration of most essential amino acids is less in pregnancies with IUGR fetuses [34], [37]. This interesting finding should be addressed in future studies.

In contrast to the reduction of the majority of the amino acid detected are the increased values of phenylalanine, a neutral branched amino acid, in LBW newborn. Higher levels of amino acids, branched and/or aromatic, have been associated with insulin resistance [38,39], and with the risk to develop type 2 diabetes in the Framingham offspring cohort [19]. The potential for amino acids to induce insulin resistance has been recognized for some time [40]. In a low-calorie environment, it is not surprising that large neutral amino acids would promote an anabolic state by inhibiting proteolysis and by directly stimulating protein synthesis [41]. That notwithstanding, the detection of significant spectral regions in the broad protein amide region (between 7.80 and 7.86 ppm) seems to support a potential difference in the anabolic rate between LBW and control newborns.

One of the greatest differences is the free choline, with low levels in the LBW group. The spectral region between 4.06 and 4.08 ppm, belonging to fragments of phosphoethanolamine [42], also shows statistically significant differences in the LBW group. During gestation there is a high demand for this essential nutrient [43]. Choline, via its metabolite betain, serves as a donor of methyl groups for the production of S-adenosylmethionine (SAM), a substrate of DNA and histone methyltransferases [44]. DNA-methylation is catalyzed by DNA methyltransferases that transfer methyl groups from SAM to cytosine located in CpG dinucleotides. Different studies have shown that the prenatal choline supply affects the expression of multiple genes whose expression is regulated via epigenetic marks [45], [46]. Maternal choline supply during pregnancy modifies fetal histone and DNA methylation in rat fetal liver and brain [47]. Conversely, animals fed with diets deficient in choline and in methionine have altered global and gene-specific DNA and histone methylation [48]. These studies have demonstrated that maternal choline alterations during pregnancy modify fetal histone and DNA methylation, suggesting that a concerted epigenomic mechanism contributes to the long-term developmental effects of varied choline intake in utero.

Finding that the lower levels of choline are associated with LBW is compatible with the epigenetic programming hypothesis. Interactions of nutrients with the epigenetic machinery lead to changes associated with the regulation of gene expression that underlies the developmental programming consequences in adult life [49]. The epigenetic programming of the fetus in utero is under the influence of the altered metabolic milieu of pregnancy and the types of nutrients available through the maternal diet [50]. Alterations in nutrients, above all those related to DNA methylation, can modify the levels of DNA methylation and fetal programming [51]. Disease propensity in LBW children can be epigenetically programmed by intrauterine exposures that have the capacity to modify fetal genes through alterations in DNA methylation [52]. Deregulation of the epigenome may explain changes that are maintained throughout adult life. Indeed, lower levels of choline can explain the link between altered epigenetic regulation and environmental influence.

The differences found in choline, proline, alanine, glutamine, glucose, citrulline and phenylalanine at birth show that metabolic alterations are present in LBW. Even though it will be interesting and useful to clarify the mechanisms by which these metabolic changes lead to LBW consequences later in life, they are difficult to establish at present. Metabolic profile may be useful, however, in detecting differential phenotypes and in identifying those populations with the greatest potential risk. The identification of these differences can facilitate the search for alterations in tracking studies. These metabolites could be promising candidates in the search for the molecular differences capable of explaining the increased risk that LBW subjects exhibit for developing cardio-metabolic diseases later in life. That notwithstanding, whether or not these abnormalities are the source of low birth weight is difficult to establish.

Our findings suggest that a substantial component of metabolic disease risk has a prenatal developmental basis. Finding discriminatory metabolites in umbilical cord blood plasma from LBW newborns with a higher risk to develop cardio-metabolic disease may lead to their being used as potential biomarkers for the early prediction of risk and with the concomitant clinical implications for follow-up.

## Conclusions

Both the presence of the differential metabolic profile, as well as the relationship of these metabolites with birth weight demonstrate that metabolic alterations are present and detectable at birth in LBW subjects. Beginning at birth, metabolic abnormalities provide information on the impact of intrauterine life. Whether these early disturbances in metabolism contribute to the development of cardio-metabolic diseases in these subjects later in life needs to be assessed in prospective studies.

## Competing interests

The authors declare that they have no competing interests.

## Authors’ contributions

EL and CI conceived the study, designed it and wrote the manuscript. CGV informed the parents of the objectives of the research project, took the anthropometric measurements at birth and obtained the UCB samples. FJC participated in the experimental design and biochemical interpretations. DM and JMM performed the NMR measurements, NMR data analysis and biochemical interpretation of metabolomic data. All authors have read and approved the final manuscript.

## Supplementary Material

Additional file 1 **Figure S1. **Loadings plot of the PLS-DA model for discrimination between spectra of umbilical cord blood plasma from low vs normal weight babies at birth. Figure S2. Loadings plot of the PLS-DA model for discrimination between spectra of blood plasma from mothers of low vs normal weight babies at birth. Click here for file
