# Supplementary material for: Metabolomic profiling in blood from umbilical cords of low birth weight newborns
Source: J Transl Med. 2012 Jul 9;10:142. doi: 10.1186/1479-5876-10-142 (PMC3551816; doi:10.1186/1479-5876-10-142)
Supplement: Additional file 1 — Figure S1. Loadings plot of the PLS-DA model for discrimination between spectra of umbilical cord blood plasma from low vs normal weight babies at birth. Figure S2. Loadings plot of the PLS-DA model for discrimination between spectra of blood plasma from mothers of low vs normal weight babies at birth. [file 1479-5876-10-142-S1.doc]

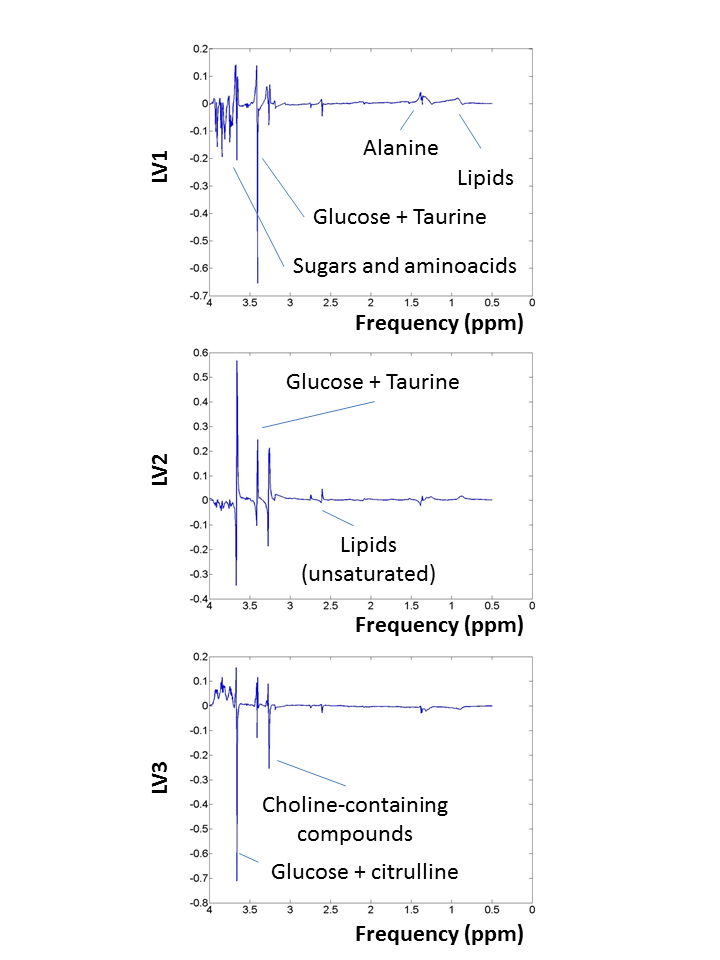


Figure S1. Loadings plot of the PLS-DA model for discrimination between spectra of umbilical cord blood plasma from low vs normal weight babies at birth.


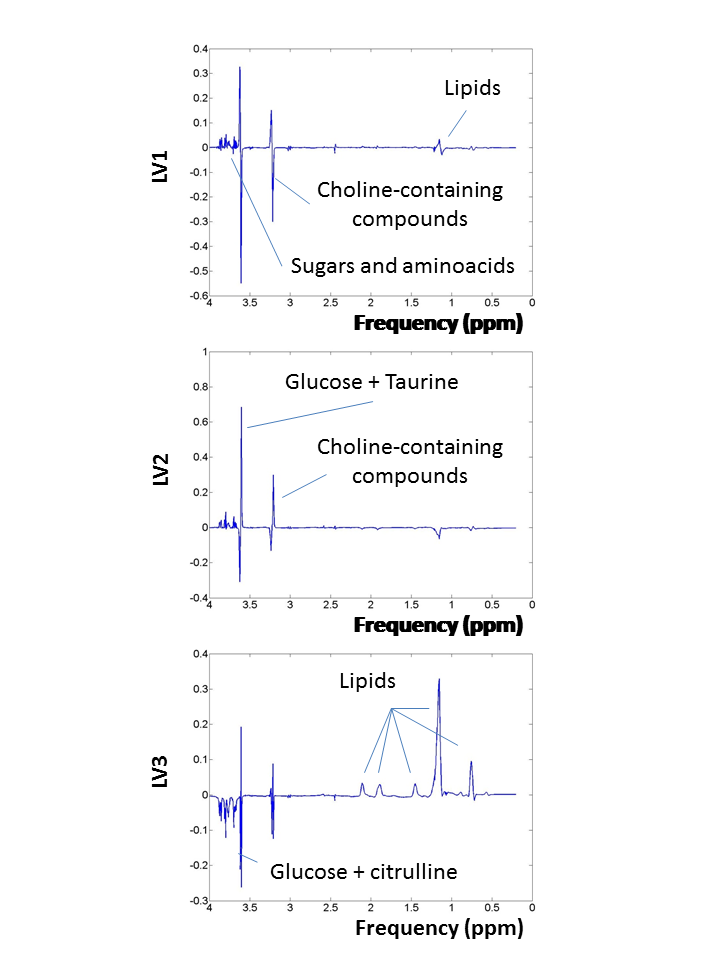


Figure S2. Loadings plot of the PLS-DA model for discrimination between spectra of blood plasma from mothers of low vs normal weight babies at birth.
